# Coming to terms with oneself: a mixed methods approach to perceived self-esteem of adult survivors of childhood maltreatment in foster care settings

**DOI:** 10.1186/s40359-018-0259-7

**Published:** 2018-09-17

**Authors:** Dina Weindl, Brigitte Lueger-Schuster

**Affiliations:** Faculty of Psychology, University of Vienna, Liebiggasse 5, 1010 Vienna, Austria

**Keywords:** Institutional maltreatment, Foster care settings, Adult survivors, Emotional self-esteem, Mixed methods approach, Thematic analysis

## Abstract

**Background:**

A broad range of psychopathological sequelae was found in adult survivors of institutional childhood maltreatment (IM). Childhood maltreatment is also associated with lower self–esteem (SE). In previous qualitative research, adult survivors of IM reported feelings of worthlessness and self-doubts, but research on IM and its associations with SE is still scarce.

**Method:**

To investigate the emotional facet of SE in 46 adult survivors of IM in foster care settings provided by the City of Vienna we used the Emotional SE subscale of the Multidimensional Self-Esteem Scale (‘Multidimensionale Selbstwertskala’, MSWS) and applied a semi-structured interview with open-ended questions. Qualitative data were analyzed with thematic analysis. Finally, qualitative and quantitative data were merged in a mixed method approach to detect similarities and differences between both assessment modalities.

**Results:**

Findings showed a significantly lower emotional SE level (MSWS) in adult survivors compared to a norm sample. Qualitative findings revealed five main themes reporting positive and negative emotions and attitudes towards oneself. Merged data showed a tendency of more positive attitudes and emotions within participants with higher emotional SE levels and more negative attitudes within participants with lower levels. No gender differences were found in both data sets.

**Conclusions:**

IM seems to predict lower emotional SE. Observed qualitative aspects of emotional SE seem to concur with symptoms of disturbances in self-organization (DSO) that are typically present in persons suffering from Complex PTSD. Considering emotional SE in future research could facilitate the understanding of the sequelae of complex trauma.

**Electronic supplementary material:**

The online version of this article (10.1186/s40359-018-0259-7) contains supplementary material, which is available to authorized users.

## Background

Various studies reported the detrimental sequelae of childhood maltreatment (CM) [1, 2]. Nevertheless, profound knowledge of consequences of interpersonal childhood abuse and neglect in foster care settings (institutional maltreatment - IM) is scarce. Following first revelations by adult survivors of IM, researchers began to investigate this worldwide phenomenon over the past ten years [3, 4]. IM comprises prolonged experiences of maltreatment (including physical, sexual, and emotional abuse, and/or physical and emotional neglect) throughout childhood and adolescence in foster care institutions [5]. It is characterized by an inappropriate use of power and authority that fails to support and potentially harms the children’s positive development and well-being [6]. In comparison to child maltreatment in familial settings, IM is often described as more severe [7], more likely to involve multiple offenders [8], and often occurring over a longer period of time [9]. Children enduring IM cannot escape from their abusive environment and social support from outside the system is lacking [10, 11]. Institutional conditions and IM support feelings of powerlessness, betrayal, and stigmatization (institutional betrayal) and disclosure of IM is hardly possible [12, 13]. Therefore, occurring symptoms are not only linked to the abusive experiences itself but also to the harmful institutional setting [14]. Adult survivors suffer from a broad range of psychopathological distress including posttraumatic stress disorder (PTSD), depression, anxiety, substance abuse, and personality disorders [15, 16]. However, consequences of IM beyond mental illness are not yet sufficiently understood but might also effect daily functioning. In the present study, we aimed to assess emotional SE in a group of adult survivors of IM with a mixed method approach.

SE is an individual’s evaluation of their qualities and self-worth [17]. It develops during childhood and typically, SE decreases during adolescence. The increasing cognitive development promotes self-evaluation based on social comparison and external feedback [18, 19]. Consequently, traumatic experiences during adolescence negatively impact SE [20, 21]. Throughout adulthood, SE increases and declines in older age (around 70) [22]. Previous research reported a gender gap throughout adulthood. Women typically show lower levels of SE than men [20, 22]. The gender gap appears to narrow down in old age [23]. However, overall SE seems to be relatively stable over lifespan [18]. High SE can serve as protective factor and helps to overcome aversive experiences [24], whereas low SE is associated with higher trauma-related stress symptoms [25]. Recently, negative self-concept as one of the Complex PTSD specific symptom dimensions of disturbances in self-organization (DSO) is included in the ICD-11 proposal for Complex PTSD [26].

Shavelson and colleagues [27] proposed a multifaceted, hierarchical model of SE, differentiating four facets: social SE, emotional SE, physical SE, and academic SE. All facets are formed by individuals’ experiences, and their interpretation of the environment and constitute a general SE factor [28]. This model received theoretical and empirical support [29]. Even though research on SE often relies on single-facet scales, e.g. Rosenberg-Self-Esteem Scale [30], a multidimensional approach may more adequately reflect characteristics and different relations of SE components to various criteria and future behavior [31–33]. Thus, the multidimensionality of SE facets should be considered in future research [17].

IM and its associations with SE, as part of psychological functioning, was hardly investigated so far. Considering the whole VIA-S sample Weindl and colleagues [34] found that IM predicted lower general SE. Previous research reported that adult survivors of IM perceived psychological strain due to limited self-related positive associations and emotions [35]. This suggests an impact of IM on the emotional facet of SE, which encompasses self-related associations and emotions, and positive or negative feelings of self-satisfaction and self-acceptance [36]. Survivors reported negative associations and emotions such as to feel too dumb to be able to reach any aim, feeling worthless, self-doubts, not believing in themselves, and depreciating themselves [35, 37]. However, these results represent the only investigations in this field and need further replication with more robust designs.

Although it is vital to understand the individuals’ emotional burden of IM, only a paucity of research considered survivors’ voices. Previous studies suggest possible repercussion of IM on the emotional facet of SE, which reflects emotional challenges survivors have to deal with. Therefore, we sought to investigate emotional SE with the help of a mixed methods approach. To our knowledge, this is the first study exploring emotional SE with two complementary approaches in a sample of adult survivors of IM: We use qualitative interviews to assess the survivors’ subjective perception of their self-worth and concurrently we use a quantitative measure of emotional SE. In the quantitative research part we used the Multidimensional Self-Esteem Scale (MSWS) [38] to dimensionally assess participants emotional SE. In the qualitative research part, we invited participants to describe situations that related to emotional SE. Based on the theoretical background, we expected to find lower levels of emotional SE in our traumatized research sample compared to the norm sample of the MSWS [38]. Assuming SE being rather stable over the lifespan [18] and considering possible gender gaps, we further expected gender differences. Namely, women showing lower levels of SE than men [20, 22], in both approaches. Finally, we compared data from both approaches. We hypothesized, that qualitative data would go beyond the dimensional measure of emotional SE in the mixed methods comparison and provide additional information on self-perception of adult IM survivors.

## Methods

### Procedure

The present study is part of the Vienna Institutional Abuse Study (VIA-S) that investigated correlates of IM in foster care settings provided by the City of Vienna [16]. Following media reports of IM in institutions operated by the City of Vienna, an independent victims’ protection organization, administered by *‘Weisser Ring - White Ring’* was established. Adult survivors who had been raised in and had experienced IM during foster care of the City of Vienna could assert their claims. Until the end of February 2016 1984 persons received compensation payments.

All participants were mainly or partly raised in institutional foster care settings provided by the City of Vienna between the late 1940s and the late 1980s. Initially, 295 persons agreed to take part in the VIA-S and 220 persons successfully completed the first part of the study (fully structured interviews + SCID I and II). For a detailed study description of the VIA-S see Lueger-Schuster et al. [16]. About one half of the participants (*N* = 104) showed interest to participate in a second, qualitative part of the study that contained open-ended questions concerning help-seeking behavior and SE, and the operant motive test (OMT). Of those, 70 participants were randomly selected and invited to take part in the qualitative in-depth interview, three to twelve months after their first interview. Finally, 46 interviews were successfully conducted at the University of Vienna. Four specialized clinical psychologists (two men and two women), experienced in clinical research and practice, conducted all interviews, with an equal number of participants respectively. To reduce feelings of discomfort and possible irritations, the participants were interviewed by the same researcher during both appointments. The study design and procedure were elaborately explained to the participants. The interviews took approximately 45 min, were audio-recorded and transcribed verbatim.

### Participants

The participants of the study sample (*N* = 46) were between 42 and 75 years old (*M* = 58.72, *SD* = 7.92) and 28.3% were female. More than a half (54.3%) were single and 45.7% were cohabiting with a partner at the time of the interview. Our study sample represent a significantly low-educated population with very restricted economical resources. In comparison to the Austrian population, the overall level of education was significantly lower [39]. Only seven participants (15.2%) were employed and the median monthly net income was €1000 (Q1 = 827: Q3 = 1612.5). Participants reported their first institutional care placement at an age between 0 and 16 with an average age of 5.5 years (*SD* = 4.3).

### Measures

#### Quantitative

To assess emotional SE quantitatively, we used the German adaptation of the Multidimensional Self-Concept Scale [40], the Multidimensional Self-Esteem Scale (Multidimensionale Selbstwertskala, MSWS) [38]. The MSWS distinguishes six facets of SE with five to seven items per facet-scale. All subscales can be applied independently. In this analysis, only the subscale ‘emotional self-esteem’ (seven items) was used. The items were rated on a seven-point scale, ranging from one (*doesn’t apply at all*) to seven (*totally applies*). Low values indicate self-doubts, self-dissatisfaction, negative attitudes, and negative emotions about oneself. Cronbach’s α for the emotional SE scale for the whole sample (*N* = 46) was α = .85.

To assess IM and intra-familial abuse the Childhood Trauma Questionnaire (CTQ) [41] was used and we computed a cumulative child abuse index for all traumatic childhood events (institutional and intra-familial) (Cronbach’s α = .90). For a detailed description, see Lueger-Schuster et al. [16].

#### Qualitative

To gain a deeper understanding of personal perceptions of emotional SE, we designed a specific semi-structured interview schedule [42] (For the detailed interview schedule please see Additional file 1). The subscale of the MSWS for emotional SE became the basis for our open-ended questions. The questions addressed (a) perceptions of self-satisfaction in accordance with other persons and (b) perceptions of dissatisfaction with oneself contradicting with those of others. The questions did not explicitly address the possibility of IM experiences affecting the participants’ lives. We conducted three pilot interviews. Afterwards we discussed concerns about the applicability, comprehensiveness and precision of the interview schedule. As a result, the wording was slightly adapted and outstanding issues clarified. All interviews started with an introduction statement *‘Now I want to ask you some questions about your self-perception*.’

### Analysis

#### Data analysis

First, we used descriptive statistical measures to outline the characteristics of the sample. Means (*M*) and standard deviations (*SD*) were used for continuous variables, and proportions were given in percent (%) for categorical variables. The data of the study sample were not normally distributed (all K-S test *p*-values < .05). Therefore, we used Mann-Whitney-U-Test for the comparison of means to evaluate the statistical significance of gender differences. We transformed *η*^*2*^ to *Cohen’s d* as effect size measure (small: around *d* = 0.2; medium: around *d* = 0.5; large: *d* ≥ 0.8) [43, 44]. We used *t*-tests to compare the results from the study sample to the norm sample of the MSWS, based on available means and standard deviations [38].

Secondly, we explored personal perceptions of emotional SE using thematic analysis (TA) [45]. The TA allows highlighting similarities and differences across the data set as well as psychological interpretation of the data. Further, it is possible to interpret both qualitative and quantitative data jointly.

**Fig. 1:**
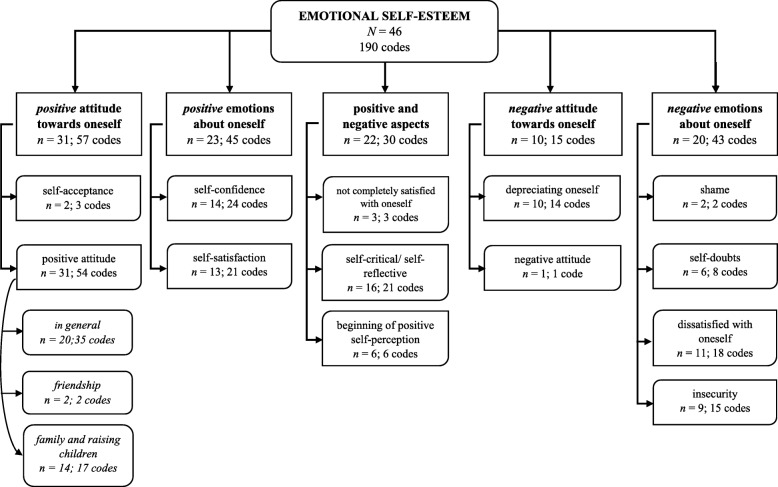
Flow chart of main and subthemes

Focusing on a detailed inquiry of possible facets of emotional SE, we assessed a top-down strategy for identifying themes within our dataset. After reading the transcripts to become acquainted with the content, two researchers developed a pre-coding frame based on two main themes distinguishing positive and negative self-perceptions. The researchers independently identified, compared, and discussed codes and themes in three randomly chosen interviews. The first author and two research assistants analyzed and discussed seven further transcripts according to the revised coding frame, agreeing on final codes and main themes. After discussion and adaptation of the coding frame, all 46 interviews were coded by the same three coders with a satisfactory level of agreement of 80.6%. To identify possible underlying subthemes, the first author and one research assistant independently revised, compared and discussed the resulting codes of the main themes on the basis of ten randomly chosen transcripts. In a final step, all interviews were analyzed following the same procedure. A detailed overview of the coding frame is provided in Fig. 1. The senior author constantly supervised the entire analysis process.

Finally, we sought to detect similarities and differences between the qualitative and quantitative datasets, and aimed to provide a broader picture of emotional SE than it would have been possible with one method only. We followed the model of Creswell and Zhang [46] merging and combining both data sets after analyzing both data sets independently. According to Creswell and Zhang [46] this merging can occur by comparing the results side-by-side (see Table 1) to examine possible similarities or contradictions. No statistical test was used within the merged data to reach further conclusions. The concurrent design provides complementary information that can be extracted from Table 1. According to the qualitative results, we sorted the participants from the lowest to the highest result in the MSWS and highlighted individuals with positive associations and emotions only.

**Table 1:** Qualitative themes and quantitative results (MSWS – emotional SE)

emot. SE MSWS^a, b^	participant	sex	Code^c^	Theme^d^	subthemes
13	P12^e^	m	2	(+) attitude	in general (2)
	P18	m	4	(+/−) aspects (−) attitude	self-critical; not completely satisfied with oneself depreciating oneself (2)
18	P15	f	11	(+) attitude (+/−) aspects (−) attitude (−) emotions	family and raising kids (2) self-critical negative attitude in general, depreciating oneself (2) insecureness (2), dissatisfied with one self (2)
	P25	f	5	(+) attitude (+/−) aspects (−) attitude (−) emotions	family and raising kids beginning of positive self -perception depreciating oneself self-doubts (2)
20	P01	f	5	(+) attitude (−) emotions	in general; family and raising kids; friendship shame, dissatisfied with oneself
21	P07	m	4	(+) attitude (+/−) aspects (−) emotions	in general self-critical shame, dissatisfied with one self
	P16	f	12	(+) attitude (+/−) aspects (−) attitude (−) emotions	in general (3) self-critical (3), beginning of positive self -perception depreciating oneself (2) dissatisfied with one self (2)
23	P19	m	2	(+/−) aspects (−) attitude	not completely satisfied with oneself depreciating oneself
	P21	m	9	(+) emotions (+/−) aspects (−) attitude (−) emotions	self-satisfaction (5), self-confidence, not completely satisfied with oneself, beginning of positive self –perception depreciating oneself, insecureness,
25	P17^e^	f	4	(+) emotions (−) emotions	self-satisfaction, self-confidence insecureness (2)
26	P02	f	5	(+) attitude (+/−) aspects:	family and raising kids; self-acceptance (2×) not completely satisfied with oneself
	P08	m	4	(+) attitude (+) emotions (−) attitude (−) emotions	in general self-satisfaction depreciating oneself insecureness
27	P40^e^	m	1	(+) emotions	self-confidence
30	P24	m	3	(−) attitude	depreciating oneself
31	P13	m	6	(+) attitude (+) emotions (+/−) aspects (−) emotions	in general self-confidence self-critical dissatisfied with one self (3×)
	P27	m	0		
33	P20	f	6	(+) attitude (+) emotions (−) emotions	friendship self-satisfaction self-doubts (2), dissatisfied with one self, self-critical,
	P23	f	2	(−) emotions	dissatisfied with one self
35	P41^e^	m	1	(+) attitude	in general
	P42	m	0		
36	P33	m	9	(+) attitude (+) emotions (+/−) aspects	in general (2), self-confidence (4), self-satisfaction self-critical (2)
38	P46^e^	m	4	(+) emotions	self-confidence (4)
39	P10	f	3	(+) emotions (+/−) aspects	self-satisfaction (2) self-critical
	P30^e^	m	3	(+) attitude (+) emotions	in general self-confidence (2×)
	P36	m	4	(+) attitude (+) emotions (+/−) aspects	in general (2) self-confidence self-critical
40	P14	m	4	(+) attitude (+) emotions (+/−) aspects	family and raising kids self-satisfaction (2) self-critical
	P45	m	2	(−) emotions	dissatisfied with one self (3)
41	P06^e^	m	2	(+) attitude	family and raising kids (2×)
	P09	m	2	(+) attitude (+/−) aspects	family and raising kids self-critical
	P22	m	6	(+/−) aspects	self-critical
	P37^e^	f	4	(+) attitude (+) emotions	family and raising kids, in general (2), self-confidence
42	P28	m	4	(+) attitude (+/−) aspects (−) emotions	in general (2) beginning of positive self -perception self-doubts
	P35^e^	m	2	(+) attitude	family and raising kids (2)
	P38^e^	m	2	(+) attitude (+) emotions	in general, self-confidence
43	P26	f	6	(+) attitude (+) emotions (−) emotions	family and raising kids self-satisfaction, self-confidence (2) dissatisfied with one self, insecureness
	P29	m	7	(+) attitude (+/−) aspects	in general (6) self-critical;
	P31	f	2	(+) attitude (−) emotions	in general insecureness
	P34^e^	m	1	(+) attitude	family and raising kids
44	P05	m	6	(+) attitude (+) emotions (+/−) aspects	self-acceptance, in general (3×), self-satisfaction self-critical
	P32^e^	m	1	(+) emotions	self-satisfaction
45	P04	f	9	(+) attitude (+) emotions (+/−) aspects (−) emotions	in general; family and raising kids, self-satisfaction, Self-confidence (2) self-critical (2) dissatisfied with one self
	P11	m	5	(+) attitude (+/−) aspects (−) emotions	in general (2) beginning of positive self -perception insecureness
48	P03^e^	m	2	(+) attitude (+) emotions	in general self-satisfaction
	P39	m	6	(+) emotions (−) emotions	self-confidence (2), self-satisfaction (3) insecureness
	P43^e^	m	5	(+) emotions	self-satisfaction (3), family and raising kids (2)
49	P44	m	4	(+) emotions (−) attitude (−) emotions	self-satisfaction (2) depreciating oneself self-doubts,
			190		

^a^raw value of the subscale emotional self-esteem; MSWS

^b^score of the subscale emotional SE – MSWS*; M* = 35.15, *SD* = 10.15; *M*_*norm*_ = 37.71, *SD*_*norm*_ = 6.76; *M*_*control*_ = 40.27, SD_control_ = 7.32

^c^number of given codes;

^d^main theme: (+) attitude *=* positive attitude toward oneself*;* (+) emotion = positive emotions of oneself; (+/−) aspects = positive and negative aspects; (−) attitude = negative attitude toward oneself, (−) emotions = negative emotions of oneself

^e^positive associations and emotions only

Verbatim transcription of the interview recordings was supported by the software f4 [47]. All interviews were conducted, transcribed, and analyzed in German language. Two researchers double-checked the transcript contents with the audio files and any information that could possibly reveal the participants’ identity was deleted. Analysis and coding of the transcripts were conducted systematically using the software ATLAS.ti 7 [48]. For the qualitative research part we followed the consolidated criteria for reporting qualitative studies [49] (see Additional file 2). For quantitative data analysis we used SPSS 22 [50].

## Results

### Quantitative analysis

Levels of emotional SE for the MSWS subscale in the study sample were significantly lower than the norms in the general population (*M* = 35.15, *SD* = 10.15; *M*_*norm*_ = 37.71, *SD*_*norm*_ = 6.76; *t*(489) = 2.32; *p* < 0.05, *d* = 0.359) (Schütz & Sellin, 2006).

### Qualitative analysis

While investigating emotional SE, 190 codes were assigned and the number of identified codes per person ranged from zero to 12 (*M* = 4.15; *SD* = 2.7). We identified five main themes: (a) *positive* attitude towards oneself, (b) *positive* emotions about oneself, (c) positive *and* negative aspects, (d) *negative* attitude towards oneself, (e) *negative* emotions about oneself (see Fig. 1). The most frequent main theme was ‘positive attitude towards oneself’ which was addressed by 31 participants, whereas a negative attitude towards oneself (*n* = 10) was the main theme least likely to be found.

#### Positive attitude toward oneself

A substantial number of participants (*n* = 31) reported positively affiliated attitudes towards themselves. Within this main theme, we identified two subthemes. First, (1) *self-acceptance* that emerged in two interviews. P2 (f, 54) reported: *‘*I finally realized that it is completely all right, if I don’t do it. And that MY perception is the most important thing. And not the other ones’. […] [I]t is easier to accept myself now, and to listen to myself. What do I want, and what do I not want*.’*

The second subtheme was an (2) *overall positive attitude*, within which three more specific themes emerged: (*i*) general attitude towards oneself, (*ii*) positive attitude towards oneself in relation to friendships, and (*iii*) positive attitude towards oneself in relation to family (life). The majority of quotes (*n* = 20, 35 codes) emerged in (*i*) the general category of ‘positive attitude towards oneself, where interviewees reported a general positive perception of themselves throughout the lifetime, such as P12 (m, 56): ‘I am proud that I always stuck to the right path, and that being honest and reliable had been very beneficial for me in the end, also with my friends.’

Participants also referred to specific incidents, in which it had been possible to experience positive perceptions of themselves: ‘… it was great (laughing), really. It showed me how much potential was inside me. It was awesome.’ (P16, f, 53).

Two participants reported a positive perception (*ii*) of friendships ‘I know that I am doing things not too badly, because I have a lot of friends that like meeting up with me (…) and then I realize that I know a lot of people who enjoy coming over for a visit.’ (P20, f, 52).

Of all participants, 14 expressed a positive perception of themselves in relation to their (*iii*) family life and children. One participant stated that giving birth to her children had been one of the few occasions where she could experience a positive perception of herself (P25, f, 57). Another participant (P6, m, 68) reported that he could recall positive self-perception when he spent time with his grandchildren: ‘Because I get the impressions that I am dealing with them appropriately.’

#### Positive emotions about oneself

Themes corresponding to positive emotions about oneself emerged in 23 participants and was divided into two subthemes: (1) *self-confidence* and (2) *self-satisfaction*.

(1) A number of participants stated that they had to fight for gaining self-confidence throughout their lives. On the other hand, they reported that no one had been able to break their self-confidence during their hard times in foster care. P46 (m, 43) called himself a ‘*skipjack’,* who expressed his opinion at all times, and P30 (m, 56) said that he is proud of not having been broken: ‘I really got beaten up by every caregiver. Even my foreman had beaten me until I was lying on the floor. But in the end they didn’t succeed. It didn’t show any effect.’ Further, participants frequently commented on not caring about other persons´ opinions and feeling confident with themselves. P4 (f, 63) stated that she did not feel the necessity to hide her past of being a ‘foster care child’ and that she had learned to stand by it: ‘I told it everyone. I stand by it, no matter what I do.’ Only one participant (P3, m, 68) reported that he associated his self-confidence with his mother who always treated him with respect and encouragement, whereas other children he knew from foster care were treated with far less.

(2) Thirteen participants reported a general feeling of self-satisfaction throughout their lifetime. P10 (f, 47) explained that she was unable to feel self-satisfaction during foster care, but felt better after leaving foster care: ‘I had never been satisfied with myself during foster care. Since the day I could direct my own life [when participant left foster care], I was feeling a lot better.’ Respondents frequently commented on specific situations in which they felt particularly satisfied with themselves, for example for having been able to stop working as a bar owner (P33, m, 54), being able to resist, being verbally aggressive, and walking away quietly (P5, m, 67), or for intervening in a difficult situation (P39, m, 73).

#### Positive and negative aspects

Another main theme identified included narratives reflecting positive as well as negative aspects of oneself. About half of the participants (*n* = 22) reported (1) self-critical/self-reflective feelings, (2) not being completely satisfied with themselves, or (3) that they were just starting to have a positive self-perception.

(1) A number of participants (*n* = 16) reflected upon their past, deeds, relationships, and themselves. Some confessed that they had been very violent in their past; P9 (m, 48) stated that only after undergoing psychotherapy he could process ‘what I [the participant] did to other people, due to my own experiences in childhood’. P7 (m, 47) considered his behavior patterns as being very narrow and perceived his surroundings’ discomfort with that matter: ‘If you really know me, you know that it has no negative effect on anyone. These are just my peculiarities, but a lot of people can’t handle them.’ Throughout the interview another participant (P18, m, 64) described having ambivalent feelings towards himself: ‘Here comes an odd balance [contradiction] that is living inside of me: my self-hatred, zero self-esteem, feeling worthless. On the flip side: pride, need for admiration, and the feeling that I am great. How does this fit together? I often keep asking myself this question.’

A few participants (*n* = 3) spoke of (2) situations and feelings in which they could feel slightly self-satisfied but nevertheless, they perceived a lack of positive emotions: ‘I had the feeling that I need to achieve it. (…) It was a nice feeling, but still not great. I have never had that.’ (P2, f, 54).

(3) Frequently, the beginning of a positively affiliated self-perception was described. Individuals talked about the changing processes of their self-perception over their life-time, into which they had put much effort. They described positive experiences, realizing that something had changed and that parts of their past were left behind.

‘I think, for myself, I have made good progress. I don’t feel so much fear anymore, and I don’t need to feel ashamed. I learned to know that I shouldn’t blame myself for all the things that have had happened (…) and that’s a good feeling. I never had that before. You often think badly about yourself, because you had been told that you were no good over such a long time.’(P2, f, 54).

#### Negative attitude towards oneself

Another main theme linked to emotional SE was negative attitude towards oneself, which was reported by one third of the participants (*n* = 15). Rather striking is the harsh and depreciating language some of the participants used when referring to themselves. They called themselves ‘asshole’, ‘loser’, and ‘failure’. Participants often expressed self-doubts. ‘I often thought that I can’t manage it. I am intellectually, psychically, vocationally not able to do it due to a lack of training and so forth. I always felt defeated’ (P22, m, 58)*.* P18 (m, 64) expressed deeply negatively affiliated attitudes that hardly left room for positive attributions: ‘In every area I’m a total “*dimwit”* who is not worth living. Self-hatred (...) calling myself “*stinking flesh*”. Nothing of me is worth anything.’

#### Negative emotions about oneself

A substantial number of interviewed participants (*n* = 20) described different negative emotions about themselves. Two participants expressed feeling of (1) shame and the fear of being pigeonholed. Therefore, they tried to hide their feelings in order to avoid being prejudged, and (2) feelings of self-doubt were identified in six narratives. They expressed grueling thoughts about own failures and worthlessness in various parts of life, and the feeling of never having had experienced positive self-perception, such as P25 (f, 57): ‘You have quite a few chances in your life, but you don’t make anything out of it, because you don’t believe in yourself. (…) After having heard that you are worth nothing for a long time, it has manifested inside yourself. You cannot get rid of it anymore. Even when you are old, you still doubt yourself.’

Further, some participants focused on general but also specific (3) feelings of dissatisfaction with oneself. Participants reported that despite their efforts they had always felt incapable of feeling satisfied with themselves: ‘I am never satisfied with myself. There is always something to find a mistake in’. Despite of being burdened by self-doubt, they were aware of the fact that satisfaction would be an appropriate feeling, as P15 (f, 55) expressed: ‘I was totally paralyzed. First, I had to digest it. In that moment, I thought that I could have been proud of myself, but I had never been. (…) I could NOT.’ Talking of specific situations, they reported feelings of having failed in giving their children what they would have had needed. They often changed jobs because they did not feel satisfied with themselves or even felt dissatisfaction because they only made the third place in a skiing race. P20 (f, 52) said what also some other participants tried to express in different words: ‘I just wanted to get away. However, I could not tell why. (…) In the end, you can go wherever you want. If you are not satisfied with yourself, you won’t be satisfied in any other place.’

Nine persons referred to feelings of (4) insecurity regarding themselves. One participant described that she always looked for jobs in big companies, so no one would recognize her. Another participant stated that she always avoided new situations and a third participant reported that she was unable to do anything on her own due to her feelings of insecurity. These feelings were explicitly linked to experiences in the past. P11 (m, 59) reported that due to these experiences he thought: ‘I am more fearful, more contemplative (…) questioning myself, scrutinizing it all.*’*

### Merging of qualitative and quantitative data

While merging both data sets, we observed a differential picture on an individual level. In the qualitative responses, positive as well as negative themes were identified in all participants regardless of their individual quantitative result. Interestingly, participants with high MSWS levels reported contrasting attitudes and emotions about themselves (e.g. P44, P39, P11) as well as participants with low MSWS levels (e.g. P18, P15, P01). Nevertheless, we detected a tendency of more positive attitudes and emotions within participants with higher levels of emotional SE, and more negative attitudes within participants with lower emotional SE levels (Table 1).

### Gender differences

We found no gender differences in MSWS scores (*U* = 169.0, z = − 1.11, *p* > .05; *η*^*2*^ = .01; *d* = .215). Similarly, we found no gender differences in qualitative data: Fisher’s exact test did not detect any significantly different frequencies for women and men (Table 2). There was a trend regarding the theme ‘negative emotions of oneself’. Women referred to more negative emotions of themselves than men (*p* = .052).

**Table 2:** Gender-specific frequencies of qualitative themes

Main theme subtheme	Men (*n* = 31)	Percent	Women (*n* = 13)	Percent
*positive* attitude towards oneself	21	67.7	10	76.9
self-acceptance	1	3.2	1	7.7
positive attitude	21	67.7	10	76.9
*in general*	*15*	*48.4*	*5*	*38.5*
*friendship*	*1*	3.2	*2*	*15.5*
*family and raising kids*	*7*	*22.6*	*7*	*53.8*
*positive* emotions of oneself	18	58.1	6	46.2
self-confidence	10	32.3	4	30.8
self-satisfaction	8	25.8	5	38.5
positive and negative aspects	16	51.6	6	46.2
not completely satisfied with oneself	3	9.7	1	7.7
self-critical/ self-reflective	11	35.5	5	38.5
beginning of positive self -perception	3	9.7	2	15.4
*negative* attitude towards oneself	7	22.6	3	23.1
depreciating oneself	7	22.6	3	23.1
negative attitude	0	0	1	7.7
*negative* emotions of oneself	11	35.5	9	69.2
shame	1	3.2	1	7.7
self-doubts	4	12.9	2	15.4
dissatisfied with oneself	4	12.9	7	53.8
insecurity	5	16.1	4	30.8

Note. Fisher’s exact test was used to test significance, no significant differences were found. No code could be assigned for two participants, reducing the total *N* to *n* = 44

Even though not statistically significant, women tended to report more positive attributions concerning family and children. Men tended to refer to rather general positive attitudes towards themselves. The subthemes ‘being dissatisfied with oneself’ and ‘insecurity’ were more often reported by women than men. No theme could be identified in the interviews of two men (P27, P42). In total, 190 codes could be identified. Of those, 74 codes (38.9%) were identified in interviews with women. This is disproportional to the ratio of women and men in the total sample of about 1:3 (Table 1).

## Discussion

The present study used a mixed methods approach to investigate the emotional facet of SE in a study sample that was highly exposed to IM during childhood and adolescence. To our knowledge, this is the first study that aimed to examine the advantages of both quantitative and qualitative data, highlighting possible associations of IM with the survivors’ emotional SE.

Results showed significantly lower emotional SE in adult IM survivors compared to the norm sample [38]. Although the observed effect was small, this finding is consistent with previous research reporting low emotional SE in other clinical samples [51, 52]. Thus, we assume that experiencing IM negatively affects self-related associations and emotions, and supports our first hypothesis. Prior research already showed that abusive experiences hinder a positive emotional perception of oneself (e.g. self-criticism [53] and support negative emotions. Individuals find themselves in a vicious circle: Their negatively toned emotional self-perception reduces their positive expectations of social reactions of their environment, establishing and maintaining close relationships becomes harder [54, 55], which again fosters negative self-perception [56]. Hence, living in an adverse environment reduces the possibility to engage in corrective experiences and to increase positive self-perception, which seems also true for the emotional facet of SE in institutional foster care settings.

Qualitative findings provided interesting insights and revealed five main themes (see Fig. 1). The negatively toned themes ‘*negative attitude towards oneself*’ and ‘*negative emotions about oneself*’ fit into the symptom dimension of ‘negative self-concept’ for Complex PTSD [57]. Knefel et al. [58] showed that the symptoms of negative self-concept together with symptoms of affect dysregulation and disturbed relationships build one cluster (disturbances in self-organization; DSO) and are proposed to represent additional symptoms for characterizing Complex PTSD, differentiating individuals suffering from PTSD from those with Complex PTSD [57]. The dimension negative self-concept contains inter alia feelings of failure, worthlessness, and shame, which were represented in the qualitative analysis of emotional SE. Further, the symptom ‘feelings of worthlessness’ occupied a central position within the network of Knefel et al. [58] and leads to the assumption that addressing emotional SE of trauma survivors in clinical treatment could also possibly ease other symptoms connected to emotional SE. Consequently, the presented qualitative data represent symptoms related to complex trauma and highlights the fact that DSO needs to be taken into account while investigating highly exposed research samples, and emphasizes the importance of past research reporting aspects of reduced emotional SE in adult survivors [35, 37].

Contrary to our assumption and prior research on SE [22], we found no significant gender differences among the MSWS scores for emotional SE. This result might be caused by a ceiling effect that relates to the harsh impact of IM, which affects women and men alike. A higher number of codes was allocated in interviews with women. In contrast, in two interviews with men we were unable to detect any theme at all. It seemed as if women potentially had easier access to reflections about their attitudes and emotions than men did. However, among men, feelings of shame and perceived threat of their masculinity may have reduced disclosure of intimate reflections of themselves [59]. Even when reporting positive attitudes towards themselves, men referred to themes that are more general whereas women seemed to be more likely to express positive attitudes related to their family and raising their children. Above all, in their responses women tended to express more self-related negative emotions and particularly discussed feelings of insecurity and dissatisfaction regarding themselves. This may be caused by the fact that women and men seem to have different gender-specific facets of their self-concept that again differently influence facets of SE. Previous research showed that boys had higher emotional stability whereas girls had higher verbal self-concepts [33].

Observing the merged data sets, participants described positive and/or negative attitudes and emotions about themselves alike across all quantitative results. Although more negatively affiliated attitudes and emotions were found in the participants with lower emotional SE scores and more positively affiliated attitudes and emotions with higher emotional SE scores, there was no clear tendency observed.

### Limitations

The major strength of this study is the mixed methods approach as it presents different perspectives of emotional SE in this specific population. Nevertheless, these results must be considered in the light of several limitations. First, we used a cross-sectional study design and thus exclude causal explanations. Second, we assume that the participants were not representative for the total population of adult survivors since we do not know whether this group was differentially adjusted than the population the participants came from. It is possible that adult survivors with poorer mental health, with significant health restrictions or without valid postal address did not find the strength or resources to participate in the study. Third, retrospective assessment of traumatic experiences and their potential consequences, as well as the assessment of subjective perceptions are vulnerable to bias. However, reviews showed that effects of retrospective assessment are negligible [60]. Fourth, quantitative and qualitative data were not collected jointly and therefore possible distortion must be considered. More than half of the participants received or still receive psychotherapeutic treatment on a regular basis. This might have interfered additionally with their views and accuracy of statements. It would be advisable to control for possible differences between treatment seekers and non-treatment seekers, but this research approach would require a larger sample size, which lies beyond the scope of this study. Besides, social desirability and a lack of introspective qualities need to be kept in mind [61]. Fifth, an evenly distributed sample according to gender or a larger sample size would potentially detect gender gaps, which could not be found in the present sample. Finally, a matched control sample would be advisable to control for possible bias, such as age, educational or economic background.

## Conclusion

This research gave voice to adult survivors of IM including their associations and emotions about themselves finding their representation in the facet of emotional SE. In sum, our data demonstrate low emotional SE in adult IM survivors. Although, women tend to express more self-related negative emotions in qualitative data no gender gaps among qualitative and quantitative data were observed. Positive and/or negative attitudes and emotions about themselves were reported alike across all participants.

Interestingly, some detected qualitative themes represents symptoms related to complex trauma. This leads to the assumption that future research on different facets of SE and their potential position in a symptom network of Complex PTSD in adult survivors could contribute to understanding the emotional burden of IM and facilitate clinical practice. It would be interesting to inspect observed dissimilarities of merged data considering possible associations with psychopathological symptoms. As symptoms of a negative self-concept were found to be central in the network analysis of Knefel et al. [58], mental disorders such as PTSD and Complex PTSD may be related to different appraisal styles.

A differentiated perspective on the facets of SE could not only help supporting clinical diagnosis, but also identifying protective factors and empowering survivors of IM in clinical practice. Furthermore, gender-specific needs and approaches need to be observed and taken into account. In the context of institutional foster care settings, an evaluation of the foster care children’s SE could facilitate a person-oriented tailored approach of supportive interventions to promote psychological functioning and well-being [24].

## Additional files

Additional file 1: Semi-structured interview schedule. (DOCX 16 kb)

Additional file 2: Consolidated criteria for reporting qualitative research (COREQ). (DOCX 22 kb)

## Abbreviations

CM: Childhood maltreatment
DSO: Disturbances in self-organization
IM: Institutional childhood maltreatment
MSWS: Multidimensionale Selbstwertskala; Multidimensional Self-Esteem Scale
OMT: Operant motive test
PTSD: Posttraumatic stress disorder
SE: Self–esteem
TA: Thematic analysis
VIA-S: Vienna Institutional Abuse Study
