# Supplementary material for: Coming to terms with oneself: a mixed methods approach to perceived self-esteem of adult survivors of childhood maltreatment in foster care settings
Source: BMC Psychol. 2018 Sep 17;6:47. doi: 10.1186/s40359-018-0259-7 (PMC6142332; doi:10.1186/s40359-018-0259-7)
Supplement: Supplementary file 1 — Semi-structured interview schedule. (DOCX 16 kb) [file 40359_2018_259_MOESM1_ESM.docx]

Additional file 1 *Semi-structured interview schedule*

|  | **Question** |
| --- | --- |
| **Introduction** | Now I want to ask you some questions about your self-perception. |
|  | Can you remember a specific situation in which you were satisfied with yourself, everything went all right, and others were satisfied with you, as well? |
|  | Can you think of a situation in which you questioned yourself or you were dissatisfied with yourself, but others expressed their satisfaction with you? |
| **In depth – questions for 1 & 2** | 1. What did you exactly think in this specific situation? 2. How did you react? 3. Can you describe it a little bit closer? 4. Did this happen more often in your life? |
| **Instruction for the interviewer** | How is the self-perception of the participant?  How does he/she describe him/herself?  How is his/her attitude towards him/herself? |
