# Supplementary material for: Coming to terms with oneself: a mixed methods approach to perceived self-esteem of adult survivors of childhood maltreatment in foster care settings
Source: BMC Psychol. 2018 Sep 17;6:47. doi: 10.1186/s40359-018-0259-7 (PMC6142332; doi:10.1186/s40359-018-0259-7)
Supplement: Supplementary file 2 — Consolidated criteria for reporting qualitative research (COREQ). (DOCX 22 kb) [file 40359_2018_259_MOESM2_ESM.docx]

Additional file 2

| No item | Description |
| --- | --- |
| **Domain 1: research team and reflexivity** | |
| 1. Interviewer/facilitator | MK, TG, VK, DW |
| 1. Credentials | All 4 interviewers: Phd students & clinical psychologist. |
| 1. Occupation | All 4 were Phd students at the university of Vienna. MK & VK were working as clinical psychologists in hospitals of the city of Vienna, TG was working as work and organizational psychologist in a consulting company and DW was working as a clinical psychologist in a foster care home in Lower Austria |
| 1. Gender | MK & TG were male, VK & DW were female |
| 1. Experience and training, relationship with participants | All four interviewers were actively working as clinical psychologist and therefore were used to assess clinical interviews. They had joined other qualitative and quantitative research projects and showed good familiarity with the assessment of qualitative interviews. |
| 1. Relationship established | There were no relationships between the interviewers and the participants. All participants were recruited with the help of the victims’ protection organization *“Weisser Ring - White Ring”.* |
| 1. Participant knowledge of the interviewer | The participants knew the interviewers from having had joined a quantitative interview some weeks before the qualitative interview. Participants knew that the interviewers are Phd students of the Faculty and Clinical Psychologists. They knew that consequences of IM were observed. |
| 1. Interviewer characteristics | The main interest of the main researcher DW was to assess the subjective perception of emotional self-esteem. |
| **Domain 2: study design** | |
| *Theoretical framework* | |
| 1. Methodological orientation and theory | Thematic analysis (Brown & Clarke)  Descriptive analysis |
| *Participant selection* |  |
| 1. Sampling | The White Ring (victims protection commission) invited all adult survivors that had received compensations payments from the city of Vienna. The signed informed consent was directly mailed to the research team. |
| 1. Method of approach | The research time got in contact with the participants per mail or telephone. |
| 1. Sample size | In the overall study VIA-S 220 participants actively joined the research project. 104 showed interest to participate in the qualitative – part, and 70 participants were randomly invited to the qualitative in-depths interview and 46 interviews were successfully conducted. |
| 1. Non-participation | 10 non- showers, 12 did not reply, 2 interviews could not be finished |
| *Setting* |  |
| 1. Setting of data-collection | All interviews were conducted at the faculty of psychology at the university of Vienna. |
| 1. Presence of non-participants | No one else was present beside the interviewers and the participants |
| 1. Description of sample | See chapter procedure & methods - participants |
| *Data collection* |  |
| 1. Interview guide | Opening questions used are shown in supplemental material B. Semi-structured interview schedule |
| 1. Repeat interviews | No repeated interviews were carried out |
| 1. Audio/visual recording | Digital audio recording |
| 1. Field notes | The researchers made field notes during and after the interviews. They included observations and personal impressions. |
| 1. Duration | Approximately 45 minutes |
| 1. Data saturation | Data saturation was discussed and achieved (no additional data was expected after the conducted interviews, due to repeated similar or recurrent themes) |
| 1. Transcripts returned | The transcripts were not returned to the participants for comments and corrections. |
| **Domain 3: analysis and findings** | |
| *Data analysis* | |
| 1. Number of data coders | 4 |
| 1. Description of the coding tree | See Figure 1 |
| 1. Derivation of the themes | See Figure 1, Table 2 |
| 1. Software | Verbatim transcription of the interview recordings was supported by the software f4. Analysis and coding of the transcripts was supported by the software ATLAS.ti 7. For quantitative data analysis we used SPSS 22 (IBM Corp., 2013). |
| 1. Participant checking | Results were presented in an informal meeting with some representatives of the participants concerned. |
| *Reporting* |  |
| 1. Quotations presented | Themes are illustrated by quotations in the result chapter. |
| 1. Data and findings consistent | From our point of view data and findings are consistent. |
| 1. Clarity of major themes | See Figure 1 and are described in the result chapter. |
| 1. Clarity of minor themes | Are described in the result chapter. |
